# Parental reports on the lexicon of children from diverse bilingual populations

**DOI:** 10.3389/fpsyg.2023.1123983

**Published:** 2023-08-03

**Authors:** Odelya Ohana, Sharon Armon-Lotem

**Affiliations:** ^1^Department of English Literature and Linguistics, Bar Ilan University, Ramat Gan, Israel; ^2^The Gonda Multidisciplinary Brain Research Center, Bar Ilan University, Ramat Gan, Israel

**Keywords:** cross-linguistic comparison, bilingualism, language exposure, parental reports, multiculturalism

## Abstract

Parental questionnaires have been widely used to assess children’s vocabularies. The MacArthur-Bates Communicative Developmental Inventories (MB-CDI) have been adapted into over 100 languages, providing researchers with access to various languages. As the vocabularies of bilingual children are distributed across their two languages, language knowledge must be assessed in both languages. While this can be done with two questionnaires, one for each language, the present study makes use of a multicultural adaptation of the MB-CDI, within a single questionnaire, that was geared specifically for bilingual context. In order to explore the developmental trajectories of the vocabularies of 90 bilingual children from diverse linguistic populations (English-Hebrew (*n* = 30), French-Hebrew (*n* = 30), and Russian-Hebrew (*n* = 30) speaking families) parents reported on both the Home Language (HL) and the Societal Language-Hebrew (SL-Hebrew). Parents also provided background information about the child, the child’s family, and exposure to each language. Our findings show no significant difference between vocabulary size of children from diverse bilingual populations in the HL and the SL, for both production and comprehension. Moreover, children from all three groups demonstrate balanced bilingualism at the group level. Correlations were found between both exposure to and use of each language by children, and various vocabulary measures across the three groups. The similar vocabulary levels demonstrated by the three groups as well as the balanced bilingualism can be explained by the relatively high prestige of all languages tested. Exposure to each language shows support in that language and a negative effect on the other language, demonstrating the crucial role exposure plays in bilingual children’s language performance.

## 1. Introduction

Bilingual children face a significant challenge when it comes to vocabulary. Multiple studies have shown that the distribution of words between the two languages is often unbalanced. This uneven distribution sometimes leads to gaps from monolingual children in at least one of the languages of bilingual children (Thordardottir et al., 2006; Miękisz et al., 2017). The challenge posed by vocabulary could be traced to its sensitivity to language contact (De Houwer, 2015), exposure variables (Armon-Lotem and Meir, 2019; Armon-Lotem et al., 2021), and literacy (Bialystok, 2002). Parental questionnaires, such as the MB-CDI (MacArthur-Bates Communicative Developmental Inventories) (Fenson et al., 1991) are often used in order to assess the lexicon of young monolingual children. For bilingual children, it has been suggested to use a questionnaire for each language in order to meet the need to assess both languages [American Speech-Language-Hearing Association (ASHA), 2004], but there are not enough assessment tools geared specifically for the bilingual population (Thordardottir et al., 2006; Boerma and Blom, 2017). Moreover, there are not many Speech and Language Pathologists who can assess bilingual children in both languages and no norms are available for bilingual children in most cases (Bedore et al., 2005; Abutbul-Oz and Armon-Lotem, 2022). In Cyprus, for example, a program for Speech and Language Therapy has only been introduced in recent years (Kambanaros and Grohmann, 2013). The multicultural questionnaire (Ohana and Armon-Lotem, 2023) has been developed to address these difficulties, in order to enable parents and Speech and Language Pathologists to report on the receptive and expressive vocabulary of bilingual children using a single questionnaire in the societal language. In the present study this questionnaire will be used to examine the effect of exposure variables such as Age of onset of Bilingualism (AOB), reported exposure to each language and its use by the child, and the effect of language of books and screens in both languages on the vocabulary of bilingual children of the three populations. Such close examination of bilingual vocabulary, comparing different populations with a single tool is expected to highlight the unique features of each population and at the same time identify commonalities in their vocabulary.

An accurate account of the vocabulary of a bilingual child should take into account both languages. This is especially important because many bilingual children are dominant in one of their languages. Evaluating bilinguals’ vocabulary in their weaker language only may result in lower vocabulary levels in comparison to monolingual children (Thordardottir et al., 2006; Miękisz et al., 2017). Moreover, bilinguals show great variability in terms of their vocabulary in each language and in both languages together, since exposure to each language and Age of Onset of Bilingualism (AOB) is different for each individual (e.g., Schwartz et al., 2009; Armon-Lotem and Meir, 2019; Thordardottir, 2019).

Due to the distribution of vocabulary across two languages, conceptual vocabulary has been proposed as a way to capture the sum of concepts known by a bilingual child. Conceptual vocabulary takes into account concepts from both languages but credits children only once for each concept they know in either one or both languages (Pearson et al., 1993). Conceptual vocabulary was found to be a good indicator of the vocabulary of bilingual children in both languages together (Junker and Stockman, 2002; O’Toole et al., 2017). A cross-linguistic study by O’Toole et al. (2017) compared vocabulary levels of bilingual children ages 24–36 months speaking a variety of languages. In their study, O’Toole et al. measured both total vocabulary and total conceptual vocabulary. They found total conceptual vocabulary a better measurement than total vocabulary when comparing different bilingual population since conceptual vocabulary reflects smaller gaps between the different versions of the CDI. Moreover, several studies show that conceptual vocabulary obtained by two independent questionnaires in the two languages of bilinguals is comparable to conceptual vocabulary obtained from a single questionnaire (e.g., Ohana and Armon-Lotem, 2023; O’Toole and Fletcher, 2010). O’Toole and Fletcher (2010) adapted the English CDI to Irish and used a single questionnaire to report on both Irish and English. They compared vocabulary levels of children on this questionnaire with direct observations of children’s vocabulary and found significant correlations between the two. Ohana and Armon-Lotem (2023) compared conceptual vocabulary that was obtained from a single multicultural questionnaire and conceptual vocabulary that was obtained from two independent questionnaires and found significant correlations between the two.

The vocabulary distribution and balance across the two languages can be influenced by the status of the acquired languages determining whether children are balanced bilinguals or dominant in one language. Languages that are spoken by a migrant minority compete with the language of the host society and their maintenance is affected by their status as well as support and acceptance by the society. In Israel, the majority of people are bilingual or multilingual, and there is a variety of languages spoken at different homes (The Central Bureau of Statistics, 2021). French and Russian are spoken in Israel by large communities, supporting their use. Both French and Russian have a relatively high status and speakers of these languages feel obliged to maintain them, and to pass them on to the next generations (Schwartz et al., 2009; Armon-Lotem and Meir, 2019). English enjoys an even higher status, being a *lingua franca* spoken by a large number of speakers, and supported by the education system in Israel (Armon-Lotem and Meir, 2019). Hebrew, as the societal language (SL), has naturally a high status and is by-and-large spoken by both monolingual and bilingual children. The high status of English and Hebrew was proposed as a possible explanation for the balanced lexicon observed among English-Hebrew bilingual children (Armon-Lotem and Ohana, 2017; Ohana and Armon-Lotem, 2023). Studies of Russian-Hebrew speakers show that despite their emphasis on HL-Russian maintenance, there is a growing trend toward SL dominance (Remennick, 2003; Altman et al., 2021). Russian in Israel has a high vitality with a large Russian speaking community that preserves Russian among the young generation. However, despite the high status of Russian for its speakers, they regard Hebrew proficiency as the key to academic success. By contrast, while English-Hebrew speakers regard Hebrew highly because of its religious and Zionist aspect, they view English as the key to academic success because of its being *lingua franca*. The different status of English and Russian, alongside the findings of the above studies, could lead to the hypothesis that the Russian-Hebrew speakers would outperform the English-Hebrew speakers in SL-Hebrew tests. A comparison of these two bilingual populations (Armon-Lotem et al., 2014), focusing on English-Hebrew and Russian-Hebrew bilingual children ages 4;4–6;1 showed, however, that the English-Hebrew speakers’ vocabulary in Hebrew was relatively the same as Hebrew vocabulary of the Russian-Hebrew group, with no significant differences between the two. To the best of our knowledge there are no recent studies investigating the vocabularies of French-Hebrew speakers in Israel.

The length of exposure to a language and the age in which language exposure begins are inherently different for monolinguals and bilinguals. For monolingual children length of exposure to their only language is identical to their chronological age since it normally begins at birth. In contrast, bilingual language exposure can begin at birth for both languages, which is the case with simultaneous bilinguals, or with exposure to one language from birth, and the other later, e.g., after the age of three for sequential bilinguals (Paradis, 2010). Some researchers distinguish bilinguals who acquire both languages from birth, from bilinguals who acquire their second language only at their second year of life, defining the latter early sequential bilinguals (Armon-Lotem et al., 2011). In both cases, language exposure is always distributed across the two languages. Therefore, aside from the length of exposure and AOB, the amount of exposure to each language must be considered when examining the vocabulary of bilingual children. Moreover, studies have shown that the amount of exposure to each language is a stronger indicator of vocabulary size than AOB and length of exposure to each language (Thordardottir, 2019).

While monolingual children receive exposure to one language only at any time and context, the language exposure of bilingual children is distributed across two languages, receiving less input than monolinguals in each (Hoff, 2018; Hoff et al., 2018). This may explain the lower vocabulary levels observed when comparing monolingual and bilingual children’s vocabulary in a single language (Thordardottir et al., 2006; Miękisz et al., 2017). Moreover, while home related concepts are often acquired in the HL, concepts related to school and to outside things may be first acquired in the SL, and only later in the HL.

Literacy and book reading at home are another important source of language exposure that must be taken into consideration when exploring the vocabulary of young children. Research has shown that exposure to literacy in a language affects vocabulary in this language positively, for both monolingual and bilingual children (Jiménez et al., 2006; Quiroz et al., 2010). Jiménez et al. (2006) investigated a sample of 16 Spanish speakers, ages 7–8 years old, exposed to English outside of their homes. Parents reported on the frequency of book reading at home, and were videotaped while engaged in book reading with their children. Their findings showed that book reading at home enhanced vocabulary in the language in which book reading was done. Another study by Quiroz et al. (2010) investigated the effect of literacy on the vocabulary of Spanish-English bilingual children ages 4–5. Quiroz et al. (2010) found that home literacy activities in one language correlated positively with vocabulary in that language and negatively with the other language. Research on the effect of literacy and book related activities at home on the vocabulary of bilingual children in other populations is rather limited, calling for further research.

It has long been suggested that parents are the best observers and reporters of the language of their children. While lab testing is limited to certain contexts and time, parents observe their children in a variety of contexts and for a lengthier period of time. Moreover, children might feel uncomfortable when tested by an experimenter, while they behave and speak freely in their natural environment (DeMayo et al., 2021). Multiple studies have used parent questionnaires that report on the vocabulary of their children, and found these questionnaires to be reliable for assessing children’s knowledge (Marchman and Martínez-Sussmann, 2002; Fenson et al., 2007; O’Toole and Fletcher, 2010), and collecting background information related to the child, his family, and language exposure patterns at home and outside of it (Schwartz et al., 2009; Armon-Lotem et al., 2014; Abutbul-Oz and Armon-Lotem, 2022). Moreover, research has shown that parents are also able to report reliably on their children’s language skills and assist in diagnosis of developmental language disorder (Auza et al., 2023).

The MacArthur-Bates Communicative Development Inventories (MB-CDI, Fenson et al., 1991) are a set of parent questionnaires allowing parents to report on the vocabulary and grammar of their children. It has been adapted to over 100 languages with several bilingual adaptations. Comparison of parental reports of their children’s vocabulary with direct measures showed that parents were able to report on their children’s knowledge accurately for both monolingual and bilingual children. Heilmann et al. (2005) tested the vocabulary of a hundred monolingual English speakers aged 30-months, with direct measures and the MB-CDI. They found significant correlations between the two, demonstrating the validity of parental reports in assessing their children’s vocabulary.

Moreover, several studies have already validated the use of a single questionnaire to assess vocabularies in both languages of bilingual children (e.g., Gatt, 2007; O’Toole and Fletcher, 2010; Dale and Penfold, 2011). For example, O’Toole and Fletcher (2010) examined the vocabulary of 21 Irish-English bilinguals aged 1;4–3;4, using a new bilingual adaptation of the CDI. They compared parent reports on vocabulary with spontaneous language samples and found significant correlations between the two. These findings validate the ability of parents to report accurately on the vocabulary of their bilingual children in both languages and to distinguish between the two languages (Marchman and Martínez-Sussmann, 2002).

With this in mind, the multicultural questionnaire used in the present study has been developed and validated (Ohana and Armon-Lotem, 2023). The multicultural questionnaire, delivered in Hebrew, the SL, includes concepts that are shared by monolingual CDI questionnaires of the SL and the HLs of the tested populations, as well as a selection of culturally specific items which are unique to the different HLs. Thus, parents report on both languages within a single questionnaire. The multicultural questionnaire was validated by comparing vocabulary levels of 38 English-Hebrew bilinguals as reported on this questionnaire with vocabulary levels as were reported for 38 English-Hebrew bilinguals on two separate questionnaires-the English CDI (Fenson et al., 1991) and the Hebrew CDI (Maital et al., 2000). Children from both groups were matched on age (24–48 months), socio economic status (mid-high SES), and age of onset of bilingualism (Mean = 4 and Mean = 4.42 for the group using the monolingual questionnaires and using the multicultural questionnaire, respectively). The study showed no effect for using two different questionnaires or a single multicultural one, no effect for language (performance on Hebrew and English were similar), with a highly significant effect for modality with comprehension higher than production across the different questionnaires. That is, parents reported similar vocabulary levels in each of the languages, independently of the questionnaires that were used for these reports. The similar responses of parents using the multicultural questionnaire to those using two separate questionnaires, support the use of a single multicultural questionnaire to report on two different languages. More details of the validation of the multicultural questionnaire are provided in Ohana and Armon-Lotem (2023).

Other studies have tested the ability of parents to report the relative exposure to each language, and other background variables that might affect children’s language performance (e.g., Hoff et al., 2012). Research has shown that parental estimation of the amount of exposure of their bilingual children to each language were accurate (Hoff et al., 2018). In a study by Place and Hoff (2011), parents were asked to report on relative exposure to each language for their bilingual English-Spanish speaking children (mean age: 25.66 months). They found that the relative exposure to each language was a significant predictor of vocabulary in that language, arguing that this demonstrated parents’ ability to report accurately on exposure to each language.

The present study uses a multicultural questionnaire (Ohana and Armon-Lotem, 2023) for evaluating the lexicon of three bilingual populations speaking Societal Language-Hebrew (SL-Hebrew) with Home Language (HL) English, French or Russian, in order to explore the differential effect of HL, language exposure and literacy exposure on the vocabulary of bilingual children in both languages.

In light of the above research, three questions will be examined next:

1. Do bilingual children, exposed to the same SL with different HLs, demonstrate different developmental trajectories of their vocabularies in each language separately and in both languages together?
2. Do reported exposure patterns (such as, reported languages spoken with the child) and reported language use (such as, reported languages used by the child) coincide with the vocabulary levels of children in each language? Is there a difference between the different HL populations?
3. How does exposure to books and screens affect vocabulary in each language? Is this effect similar across the HL groups?

The following hypotheses are tested:

1. The developmental trajectories in each language separately and in both languages together are hypothesized to reflect their status and vitality within each community. It is predicted that bilingual children exposed to English, Russian, or French at home, with SL-Hebrew are expected to demonstrate balanced bilingualism as a group. This expectation for balanced bilingualism is due to the intense exposure children receive to both Hebrew and the HLs. While Hebrew is the SL, supported by the educational system, has a religious prestige and often viewed as key to integration in society, the three HLs enjoy a high status, dense communities, and high maintenance and support within the home and community. These large communities view their HLs as means for communicating with transnational family and preserving their homeland culture. English speakers are expected to present an advantage in their HL vocabulary over French and Russian speakers, since English is also a *lingua franca* with an academic value supported by the education system in Israel.
2. It is hypothesized that the amount of language exposure impact vocabulary size (Hoff et al., 2012). That is, the more children are exposed to one language, the higher their vocabulary should be in that language. Thus, we predict that reported exposure to SL-Hebrew is expected to have a positive effect on reported vocabulary in Hebrew and a negative effect on vocabulary in the HL. That, is, parent reports of languages spoken by the child are expected to be consistent with reports on vocabulary in each language.
3. Exposure to books and screens in one language is expected to correlate positively with vocabulary in that language and negatively with vocabulary in the other language (e.g., Quiroz et al., 2010).

## 2. Materials and methods

### 2.1. Participants

Data were collected from parents of 90 bilingual children, aged 24–48 months: 30 English-Hebrew speakers (15 girls) (M = 37.63, SD = 8.87), 30 French-Hebrew speakers (15 girls) (M = 37.60, SD = 8.02), and 30 Russian-Hebrew speakers (14 girls) (M = 37.57, SD = 9.20). All children were either simultaneous bilinguals from one-parent-one-language homes or early sequential bilinguals who were exposed to their second language before the age of two, acquiring the HL at home and the SL-Hebrew, outside of their homes. Most children (*n* = 80) were attending a day care where the SL-Hebrew was used. Children had at least 6 months of exposure to the SL-Hebrew, similarly to the threshold determined in previous studies (e.g., O’Toole et al., 2017). Most children come from mid-high SES with parents who have an academic degree or at least a professional certification. Aside from one family from the French-Hebrew speaking group where both parents are unemployed, and seven families out of the three groups, where one parent is reported to be unemployed, all other parents are employed and several others are enrolled in academic studies. Table 1 presents background information for the entire sample (*N* = 90).

**Table 1:** Background information (*N* = 90).

		English-Hebrew	French-Hebrew	Russian-Hebrew	Statistics
Number (females)		30 (15)	30 (15)	30 (14)	
Age in Months	Mean	37.63	37.60	37.57	*F* (2,87) = 0.000
	SD	8.87	8.02	9.20	
	Range	24–48	24–48	24–48	*p* = 0.816
AOB (in months)	Mean	4.62	7.88	9.41	*F* (2,85) = 1.77
	SD	10.01	8.74	11.37	
	Range	0–40	0–33	0–36	*p* = 0.176
Family size* (no. of children)	1–2	13	18	25	*X*^2^ (8, *N* = 90) = 15.73
	3–4	9	6	2	
	5+	5	5	3	*p* = 0.046
Birth order	First born	13	13	20	*X*^2^ (6, *N* = 90) = 8.05
	Second born	6	4	5	
	Later born	11	12	5	*p* = 0.235
Family Income**	> average	15	12	19	*X*^2^ (6, *N* = 90) = 21.41
	= average	7	6	11	
	< average	5	10	0	*p* = 0.002
Mother Education***	Academic/professional	25	25	29	
	High school graduate	2	3	1	
	Elementary/none	0	1	0	
Father Education***	Academic/professional	23	20	23	
	High school graduate	4	4	4	
	Elementary/none	0	3	0	

*Data about family size is missing for three participants in the English-Hebrew group and for one child in the French-Hebrew group. **Data about family income is missing for three participants in the English-Hebrew group and for two participants in the French-Hebrew group. ***Data about parents’ education is missing for six participants in the English-Hebrew group, for four participant in the French-Hebrew group, and for three participants in the Russian-Hebrew group. AOB-Age of onset of bilingualism – the age in which exposure to SL-Hebrew has started.

No significant between-group differences were observed for the chronological age of the children, and the AOB and onset of exposure to the HL. In terms of family size, the majority of Russian-Hebrew speakers come from small families with one or two children, whereas in the English-Hebrew and the French-Hebrew speakers about a half of the group reports on three or more children. A chi-square test of independence showed there was a significant association between group and family size, due to significant difference between the English and the Russian cohort (*p* = 0.006), but no significant association between group and birth order. For family income, parents reported whether the family income is average, below, or more than the average. Family income was found to have a highly significant association with group, showing the following hierarchy: Russian>English>French.

### 2.2. Instruments and procedures

**Figure 1:**
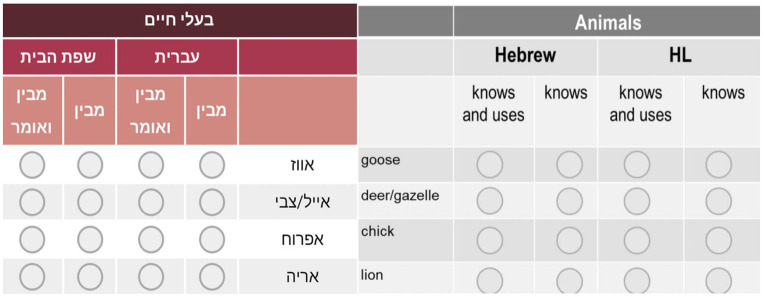
A part of the multicultural questionnaire with its translation* (four concepts from the category of animals’). *The questionnaire was in Hebrew only. The English is a translation for the benefit of the reader.

The vocabularies of bilingual children were reported using a multicultural questionnaire (see Figure 1) that enabled parents to report on the vocabularies of bilingual children in both the HL and the SL-Hebrew with a single questionnaire in the SL-Hebrew (Ohana and Armon-Lotem, 2023). The multicultural questionnaire is an adaptation of the Hebrew CDI-Words and Sentences (HCDI: WS) (Maital et al., 2000), which originally consisted of a list of 602 items. From this list, three irrelevant items were removed (e.g., tape/cassette). To these, 34 culturally specific words selected from the English, Russian, and French CDI questionnaires were added, and three were removed, resulting in a list of 633 items (Fenson et al., 1991; Kern, 2007; Vershinina et al., 2011). These items were added mainly, but no only, to the category of Foods and Drinks (e.g., peanut butter, cabbage and baguette, from the English, Russian, and French CDIs, respectively). The selected items were added to the questionnaire in consultation with groups of parents from each bilingual population. Each group of parents was presented individually with the Hebrew adaptation of the CDI, along with the CDI version of their HL. The parents explored both questionnaires and pointed to relevant concepts that were found on the CDI in their HL, and were missing on the Hebrew CDI. These items were added in order to capture concepts in use by children from different homes and cultures, making the multilingual questionnaire a valid tool for the assessment of bilingual children. This resulted in a list of 633 concepts in the SL-Hebrew, divided into categories (such as, animals, people etc.). For each concept, parents indicate whether their child knows this concept in the HL and/or the SL-Hebrew, addressing both comprehension and production. Parents also completed a background information form (Gendler-Shalev and Dromi, 2021) which included general information regarding language exposure patterns, child’s developmental milestones, as well as information about the parents and the family. Participant recruitment was done through the social media, through groups of speakers of languages other than Hebrew in Israel, and by word-of-mouth. Parents used a link to the home page of the study to complete the questionnaire at their own convenience.^1^ Once parents completed a short registration form, and gave their consent to participating in the study, they were transferred directly to the questionnaire. A full account of the procedures of creating the multicultural questionnaire is provided in Ohana and Armon-Lotem (2023).

### 2.3. Data analysis

The number of words comprehended and produced in each language on its own, and the conceptual vocabulary from both languages were calculated for each child automatically. Data analysis was performed using the IBM SPSS Statistics. Analyses included a three way – ANCOVA for repeated measures with Group (English, French, Russian), Language (HL, SL-Hebrew), and Modality (production, comprehension) as the independent variables, vocabulary levels as the dependent variable, and Age and Age of Onset of Bilingualism (AOB) as the covariates. A separate two-way ANOVA was computed for the conceptual vocabulary, with Group and Modality as the independent variables.

Further analyses were conducted to explore the effect of exposure variables on the individual vocabulary levels and to determine whether there is a match between reports of the two. Exposure to the SL-Hebrew and the use of SL by the child were reported on a 1–7 Likert scale (1. Only HL, 2. 2 h of Hebrew every day, 3. 4 h of Hebrew, 4. 6 h of Hebrew, 5. 8 h of Hebrew, 6. 10 h of Hebrew, 7. Only Hebrew). Under the assumption that children aged 2–4 years old have around 12 waking hours, children with reported 6 h of Hebrew per day were defined as children with equal exposure to both languages (HL = SL), whereas less than six hours of exposure to Hebrew is defined as dominant exposure in the HL (HL > SL) and more than six hours of Hebrew is defined as dominant exposure to SL (SL > HL).

Correlational analyses were computed to test exposure variables such as, chronological age, and AOB, on exposure and use of each language by the child, and their relation to vocabulary reports. In addition, for each child the gap between both languages was calculated (i.e., the number of words in HL minus the number of words in SL) and its relation to exposure variables was examined, in order to explore the effect of exposure on language dominance. A positive score indicates a larger vocabulary in HL in comparison to SL and vice versa.

Finally, exposure to books and screens was tested in order to evaluate their relative contribution to vocabulary levels in each language. Exposure to books was reported on a 1–4 Likert scale (1. rarely; 2. 1–2 times a week; 3. 3–5 times a week; 4. At least 1 book every day). Exposure to screens was reported on a 1–5 Likert scale, indicating the relative exposure to screens every day (1. no exposure, 2. rarely, 3. 1 h per day, 4. 2 h per day, 5. 3 h or more every day). Language of books/screens was reported on a Likert scale of 1–3 indicating the language in which books are read (1. Mainly in the HL, 2. Equally in both languages, 3. Mainly in the SL-Hebrew). Correlational analyses followed by hierarchical regression analyses were performed on the data to explore the effect of frequency of exposure as well as the language in which children were exposed to books and screens. Language of screens was entered into the regression as a variable determining the amount of Hebrew exposure through books and screens. Low Hebrew exposure means higher HL exposure through books and screens since exposure was reported on a scale between reading/watching only in Hebrew, in both language or only in HL without taking frequency into consideration. Separate hierarchical regressions were performed for each group, for both vocabulary production and vocabulary comprehension as the dependent variables, and age and exposure to books and screens as the predictors. Age was entered into each regression in the first step, and exposure to books and screens were entered in the second step to explore their effect on vocabulary size beyond children’s age.

## 3. Results

To address the above questions, we start by comparing vocabulary size for the three groups and commence with an exploration of the relation between exposure and background factors and vocabulary size.

### 3.1. Vocabulary size: by group

Descriptive statistics for the vocabulary of the entire sample are presented in Table 2, for both languages of English-Hebrew, French-Hebrew, and Russian-Hebrew speakers, for both production and comprehension (*N* = 90).

**Table 2:** Vocabulary levels of English-Hebrew speakers, French-Hebrew speakers, and Russian-Hebrew speakers in each language and in both languages together (conceptual vocabulary).

		English-Hebrew (*n* = 30)	French-Hebrew (*n* = 30)	Russian-Hebrew (*n* = 30)	Statistics
Home language mean (SD)	Production	443.37 (199.68)	415.47 (198.90)	443.00 (216.60)	Group: *F* (1, 86) = 0.079, *p* = 0.924, $\eta_{p}^{2}$ = 0.002
	Comprehension	556.03 (115.15)	541.07 (140.53)	529.60 (148.34)	Modality: *F* (1,87) = 101.316, *p* < 0.001, $\eta_{p}^{2}$ = 0.538
SL-Hebrew mean (SD)	Production	415.43 (200.63)	451.97 (184.95)	436.87 (197.41)	Language: *F* (1,87) = 0.004, *p* = 0.950, $\eta_{p}^{2}$ <0.001
	Comprehension	541.30 (123.00)	538.53 (167.19)	527.07 (158.73)	
Conceptual vocabulary mean (SD)	Production	517.77 (154.23)	533.43 (120.78)	515.63 (175.83)	Group: *F* (1,87) = 0.099, *p* = 0.906, $\eta_{p}^{2}$ =0.002
	Comprehension	578.37 (84.31)	589.37 (87.14)	563.20 (120.14)	Modality: *F* (1,87) = 41.584, *p* < 0.001, $\eta_{p}^{2}$ =0.323

A Three-Way Mixed ANCOVA with Group, Language, and Modality (production/comprehension) as independent variables, vocabulary levels as the dependent variable, and age as a covariate, shows that there is no main effect of Group, with all groups performing similarly overall. Moreover, independently of the group tested, there is a highly significant main effect for Modality, with comprehension rates higher than production rates. Additionally, no effect for Language was found, showing that children demonstrated similar vocabulary levels in both the HL and SL-Hebrew, with no significant differences between the two across the entire sample.

For the conceptual vocabulary, ANOVA performed on the data revealed no main effect of Group, with all three groups showing similar conceptual vocabulary levels for both production and comprehension. Similarly to results in each language separately, conceptual vocabulary demonstrates a significant main effect for Modality, with comprehension rates significantly higher than production rates. These findings remained consistent when controlling for age.

### 3.2. Vocabulary size: individual scores

**Figure 2:**
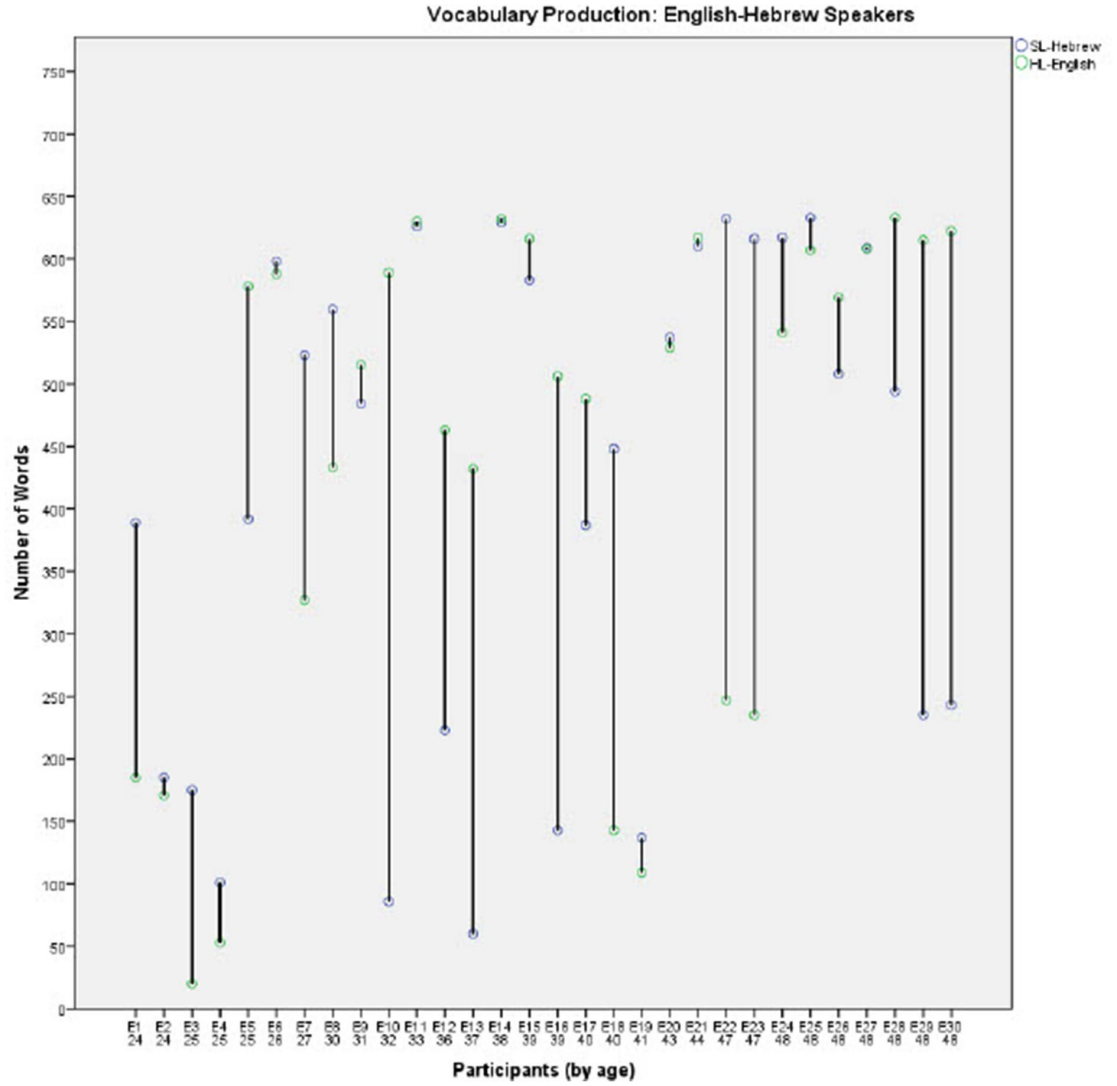
Vocabulary production levels for the English-Hebrew speakers.

**Figure 3:**
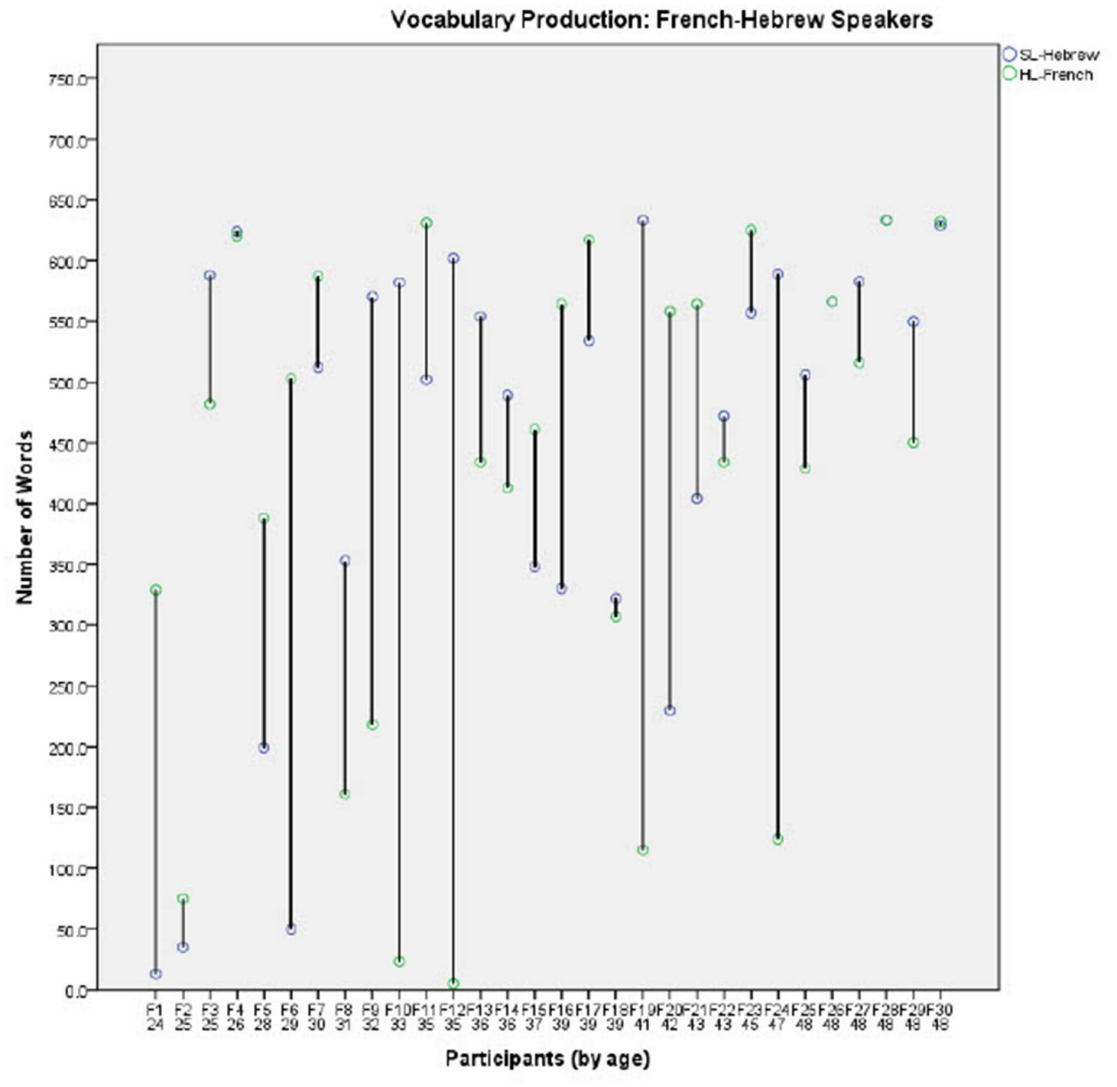
Vocabulary production levels for the French-Hebrew speakers.

**Figure 4:**
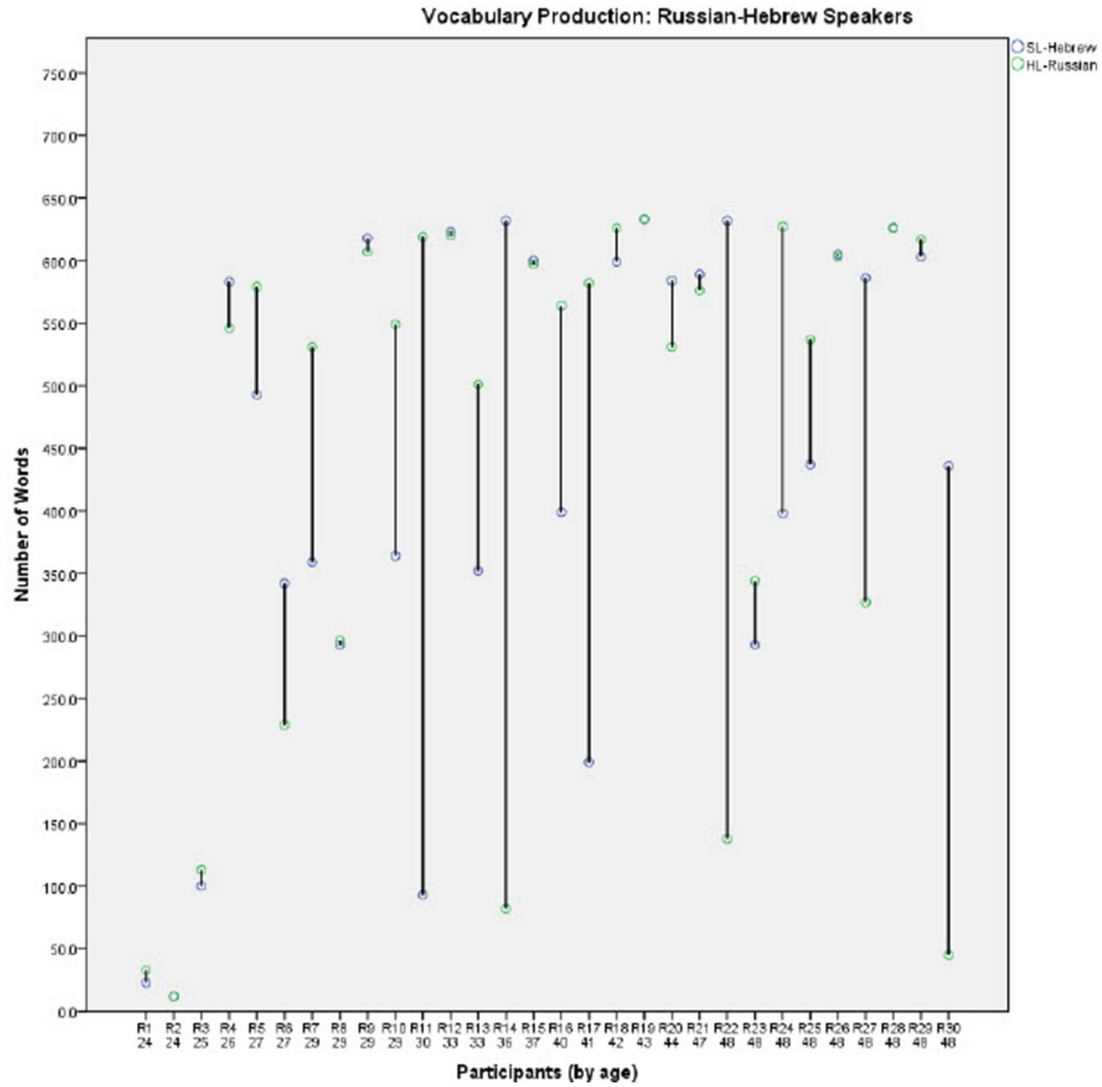
Vocabulary production levels for the Russian-Hebrew speakers.

In order to further investigate the effect of Group on the developmental trajectories of each language, comparisons of the individual vocabulary production scores in the two languages are presented in Figures 2–4 for each group separately (for the English-Hebrew, French-Hebrew, and Russian-Hebrew groups, respectively). Each figure presents the number of concepts each child produces in both the HL and the SL- Hebrew, for the 30 participants in the group. Since the multicultural questionnaire is in the SL-Hebrew each concept represents two words, one in SL-Hebrew and the other one in the HL. The participants are presented in ordinal numbers with a capital letter representing their HL (for example, E1 represents participant 1 in the English-speaking group, E2-English participant 2, F1-French speaking participant 1, R1-Russian speaking participant 1 etc.). Under each participant’s number, the age of the child is provided. For each participant, two data points are presented, for vocabulary in the HL and the SL-Hebrew.

Figures 2–4 illustrate the great variability between individuals, within the three groups. Some children demonstrate similar vocabulary levels in both the HL and the SL-Hebrew, and many others are highly dominant in one of their languages. A close inspection of individual children suggests that the amount of exposure to each language reported for individual children could be a possible source of the large gaps some children have between their vocabularies in the HL and SL-Hebrew. For example, E30 in the English-Hebrew sample (Figure 2) shows a great dominance in English over Hebrew, and this report is consistent with reported exposure patterns as parents reported that their child hardly speaks Hebrew. Another example is shown by F24 in the French-Hebrew sample (Figure 3) and R30 in the Russian-Hebrew sample (Figure 4) who both show a great advantage of vocabulary in their SL-Hebrew over their HL, and for both parents reported that they speak only in Hebrew and not in the HL. Thus, we next turn to the relation between exposure patterns and vocabulary size.

### 3.3. Language exposure and use

Table 3 presents AOB, language exposure by others, and language use by the child, for all three groups, providing the number of participants for the different patterns of exposure and use.

**Table 3:** Exposure variables by number of participants for each population.

		English-Hebrew	French-Hebrew	Russian-Hebrew
AOB (in months)	Mean	4.62	7.88	9.41
	SD	10.01	8.74	11.37
	Range	0–40	0–33	0–36
Language exposure (by others)	HL > SL	4	5	9
	HL = SL	8	10	2
	HL < SL	18	15	19
Language use (by the child)	HL > SL	9	8	7
	HL = SL	3	3	4
	HL < SL	16	15	19

AOB, Age of Onset of Bilingualism refers to the age in which exposure to SL-Hebrew has started, HL > SL – up to 4 h of Hebrew, HL = SL – 6 h of Hebrew, HL < SL – 8 or more hours of Hebrew.

Table 3 shows that across the three groups, all the participants were exposed to the SL-Hebrew in the first year of life. Moreover, about half of the participants in each group were exposed to and used SL-Hebrew more than their HL.

Table 4 demonstrates that SL-exposure presents limited correlation with both HL or SL-vocabulary production (apart from a negative correlation with HL-vocabulary production among English-Hebrew speakers), while significant correlations were observed between HL- and SL-vocabulary production and language use by the child. Since the language directed at the child did not correlate with language outcomes, we next turn to investigate the relation between the language used by the child and vocabulary measures. SL use by the child correlated negatively with HL-vocabulary production scores for the English-Hebrew and Russian-Hebrew speaking populations but not for the French-Hebrew speaking population, while SL use by the child showed positive correlations with SL-vocabulary production across the three groups. The gap between the HL and the SL (HL minus SL), for both production and comprehension, strongly correlated with both language exposure and language use by the child (apart from the correlation between exposure and production gap for the Russian-Hebrew group). As a negative gap score indicates Hebrew dominance, the negative correlations indicate that with more exposure to SL-Hebrew children become more dominant in Hebrew, and being more dominant in Hebrew they use it more and understand it better.

**Table 4:** Correlation between exposure to and use of Hebrew by the child and the gap between the two languages.

	SL-Hebrew exposure			SL-Hebrew used by the child		
	En-Heb	Fre-Heb	Rus-Heb	En-Heb	Fre-Heb	Rus-Heb
HL-production	−0.433*	ns.	ns.	−0.554**	ns.	−0.421*
SL-production	ns.	ns.	ns.	0.397*	0.522**	0.593**
ProdGap	−0.703**	−0.540**	ns.	−0.823**	−0.689**	−0.754**
CompGap	−0.566**	−0.368*	−0.453*	−0.598**	−0.429*	−0.689**

*p < 0.05, **p < 0.01. ProdGap and CompGap refer to the gaps between words in the two languages (HL-SL) for both production and comprehension. The larger the gap between the languages is, the better children are on the HL in comparison to the SL-Hebrew.

### 3.4. Exposure to books and screens

We next turn to the effect of books and screens on the vocabulary of children in each language. Table 5 presents reports on the language exposure by books and screens, for each group, by the number of participants. Each row indicates the number of participants with each exposure pattern.

**Table 5:** Reported language of books and screens for each group.

		English-Hebrew (number of participants)	French-Hebrew	Russian-Hebrew	Statistics
Language of Books	HL > SL	13	8	10	*X*^2^ (4, *N* = 89) = 6.26
	HL = SL	11	7	12	
	HL < SL	6	14	8	*p* = 0.181
Language of screens	HL > SL	19	10	13	*X*^2^ (4, *N* = 77) = 7.87
	HL = SL	6	7	10	
	HL < SL	1	6	5	*p* = 0.096

HL > SL – up to 4 h of Hebrew, HL = SL – 6 h of Hebrew, HL < SL – 8 or more hours of Hebrew.

A chi-square test of independence showed there was no association between group and both language of books (*X*^2^ (4, *N* = 89) = 6.26, *p* = 0.181) and language of screens (*X*^2^ (4, *N* = 77) = 7.87, *p* = 0.096). Further correlational analyses were performed to examine the effect of language of books and screens on vocabulary levels. Table 6 presents correlations between amount of Hebrew in books and screens and vocabulary in each language for both production and comprehension.

**Table 6:** Correlation between exposure to Hebrew in books and screens and vocabulary levels in both Hebrew and the HL.

	SL-Hebrew exposure in books			SL-Hebrew exposure in screens		
	En-Heb	Fr-Heb	Rus-Heb	En-Heb	Fr-Heb	Rus-Heb
HL-Prod	−0.453**	ns.	ns.	−0.326	ns.	ns.
HL-Comp	−0.461**	ns.	ns.	−0.380*	ns.	ns.
SL-Prod	ns.	0.397*	ns.	ns.	*r* = 0.337	ns.
SL-Comp	ns.	*r* = 0.399*	ns.	ns.	ns.	ns.

*p < 0.05, **p < 0.01.

Exposure to the SL-Hebrew in books and on screens was not consistent across the three groups. For the English-Hebrew speaking group, SL-Hebrew exposure in both books and screens correlated negatively with both vocabulary production and comprehension in the HL-English. All these correlations were significant aside from the negative correlation between SL-Hebrew exposure to screens and vocabulary production in the HL-English which was nearly significant. For the French-Hebrew speaking group SL-Hebrew exposure in books correlated positively with both vocabulary production and comprehension in the SL-Hebrew. In addition, SL-Hebrew exposure in screens correlated positively with vocabulary production in this language, but not with vocabulary comprehension. For the Russian-Hebrew speaking group no correlations were found between SL-Hebrew exposure in books and screens and any of the vocabulary measures.

Hierarchical regression analyses were performed to further explore whether the effect of language exposure in books and screens on vocabulary size in each language goes beyond the age effect. Four separate Hierarchical regression models were conducted for each group, in each language, for both production and comprehension (for example, for the English-Hebrew speaking group there were separate models for English production, English comprehension, Hebrew production, Hebrew comprehension). In each model, age was introduced in the first step. Both language of books and language of screens were added, as two separate variables, in the second step. Vocabulary was the dependent variable. Results are presented in Table 7. Across the three groups only five models were significant and are presented in the table.

**Table 7:** Statistical reporting (including Δ*R*^2^ and Δ*F*) of Hierarchical regressions with age and exposure to books and screens predicting vocabulary size across the three groups.

	English-Hebrew speakers				French-Hebrew speakers		Russian-Hebrew speakers			
	English Production		English Comprehension		Hebrew Comprehension		Hebrew Production		Hebrew Comprehension	
	Estimate	*t*	Estimate	*t*	Estimate	*t*	Estimate	*t*	Estimate	*t*
Step 1: age										
Age	4.51	2.146*	2.54	1.89	4.37	2.22*	3.67	2.885**	2.62	3.47**
*R*^2^	0.161		0.13		0.19		0.24		0.32	
*F*	4.60*		3.57		4.94*		8.32**		12.03**	
Step 2: exposure										
Age	4.12	2.13*	2.29	1.86	4.23	2.19*	3.59	2.95**	2.72	3.37**
Language of screens	69.78	−0.77	38.76	−1.11	48.39	0.54	47.23	1.11	35.68	0.55
Language of books	47.79	−1.97	26.55	−1.89	45.66	1.20	46.83	0.79	35.38	0.08
Δ*R*^2^	0.36		0.36		0.331		0.334		0.33	
Δ*F*	4.13*		4.08*		3.13*		4.01*		3.92*	

*p < 0.05, **p < 0.01. Only significant models are presented.

In the English-Hebrew group, both models which predict vocabulary production in English were significant. The model with exposure to books and screens together with age explained 36% of the variance in vocabulary size. While age made a significant contribution to the model, and exposure to books made a marginal contribution to the model, exposure to screens did not make a statistically significant contribution to the model. Likewise for comprehension, the second model, where age is combined with exposure to books and screens explained 23% of variance, beyond the 13% of the variance which was explained by age only in the first step. Overall, the regression explained 36% of the variance. Only the contribution of exposure to books contributed to the explained variance of English comprehension beyond age and the exposure to screens was not a significant predictor. Similar results were found for the Russian-Hebrew speaking group. Both models predicting Hebrew vocabulary production were significant. The first model with age as the only predictor explains 24% of the variance while the second model explains 33%. For comprehension both models were significant but account for relatively the same variance in vocabulary size (32% and 33% for the first and the second models, respectively). Interestingly enough, in the French-Hebrew speaking group a similar picture was revealed only for the models predicting Hebrew vocabulary comprehension which were both significant. While the model with age as the only predictor accounts for 19% of the variance, the model with age combined with exposure variables explains 33% of the variance.

## 4. Discussion

The present study aims at identifying the developmental trajectories of the vocabularies of children from three bilingual populations, English-Hebrew, French-Hebrew, and Russian-Hebrew speakers. Our finding for each research question will be addressed separately.

### 4.1. Vocabulary level in both HL and SL-Hebrew

Our findings show that, bilingual children speaking English, French, and Russian as the HL and Hebrew as the SL have similar vocabulary levels in each language on its own and in both languages together (Tables 2, 3). The similar vocabulary levels can be attributed to the characteristics of these three bilingual populations. All three populations are exposed to SL-Hebrew in daycare centers and preschools, and very often at home as well. On the other hand, aside from the fact that English is a *lingua franca*, all three languages are widely spoken and by large communities which support and strengthen the use of the HLs. As expected, (Ring and Fenson, 2000; Abdelwahab et al., 2021) parents report significantly higher comprehension rates than production rates across the three groups, independently of the language examined. This is also true for the conceptual vocabulary which represents vocabulary from both the HL and SL-Hebrew. The ability of parents to distinguish comprehension from production is documented in the literature (Ring and Fenson, 2000). Moreover, though children demonstrate balanced bilingualism at the group level, there is a great variability within the group. Individual results show that some children demonstrate balanced bilingualism with similar vocabulary levels in both languages, but many others demonstrate dominant bilingualism with large gaps between their reported vocabulary in the two languages, demonstrating dominance in either the HL or SL-Hebrew. This variability is shown by the large SDs presented for each bilingual group and is in line with reports from previous research (Fenson et al., 2000; Armon-Lotem and Meir, 2019; Frank et al., 2021).

### 4.2. Language exposure

To further understand the great variability within the groups and the factors affecting language dominance, exposure patterns to each language were investigated. Across the three groups, children showed very similar AOB ranges, similar patterns of exposure to each language by others, and similar language use by the child. AOB did not correlate significantly with vocabulary measures in all three groups apart from a correlation with the gap between the HL and SL vocabulary within the English-speaking group. This correlation can be explained by the characteristics of English-speaking homes and the prestigious status of English which enable parents to maintain exposure to HL-English until children are officially exposed to the SL-Hebrew. It is important to note, that for all three populations the majority of children were attending a Hebrew speaking day-care that usually starts at the age of 3.5–6 months, and therefore they showed balanced bilingualism as a group. Moreover, most of the children were exposed to the SL-Hebrew before the age of 6 months, and many were exposed to the SL-Hebrew from birth. This could explain the lack of correlation between AOB and vocabulary measures. Furthermore, previous studies found AOB is not a strong enough predictor of vocabulary since it provides information about the starting point, and the length of exposure to the SL-Hebrew but not the amount of exposure. The amount of exposure to each language was found to be a better predictor of vocabulary size than the length of exposure to each language (Thordardottir, 2019). Children with the same AOB and length of exposure can still vary on the actual exposure they get to the SL-Hebrew (Armon-Lotem and Meir, 2019).

In line with findings from the literature, there is a relation between parent reports on vocabulary size and reports on both exposure by others and use of SL-Hebrew by the child. SL-Hebrew use by the child correlated significantly with vocabulary production in Hebrew across the three bilingual populations and negatively with vocabulary production in the HL for the English and Russian speaking groups. The lack of correlation between child Hebrew use and the HL vocabulary production score of the French speakers could reflect the recency of this migration and the enclaved neighborhoods of French speakers, where French is supported outside the homes and not just in the home. Exposure to SL-Hebrew mostly does not correlate with the production of either HL or SL-Hebrew, but rather with the gap between the two languages. This shows that the more exposure a bilingual child receives to one language, the more he/she uses that language, and achieves higher vocabulary levels. Exposure, use and higher vocabulary levels in one language, inevitably reduce vocabulary levels in the other language.

### 4.3. Exposure to literacy and screens

A differential effect was observed for exposure to books and screens in Hebrew. For English speakers, a negative relation was observed with English production and comprehension, while being not significant for Hebrew. Among the French-Hebrew speakers, exposure to Hebrew in books and screens is related to better Hebrew production and comprehension with no impact on HL-French. The different patterns for English speakers and French speakers might reflect the observed difference in the preference of reading, with English speakers reading more in HL than SL, and French speakers reading more in SL than HL, as well as the value attributed by the two populations for integration within the host society and academic system. With English being *lingua franca* supported in schools and academic studies, its speakers support the literacy in this language (including pro HL reading practices), while for French speakers, SL-Hebrew literacy is a key to academic integration. These observations require further research to test this hypothesis. Finally, for the Russian-Hebrew speaking group no effect was found for books and screens, perhaps due to the similar exposure received through these means for both the HL-Russian and the SL-Hebrew. These findings are in line with results from previous studies demonstrating the positive effect of book reading on the language in which reading is done, and the negative effect on the other language (Jiménez et al., 2006; Quiroz et al., 2010). Interestingly enough, regression analyses showed that while age explains relatively small portion of the variance in vocabulary size, across the three groups, the combination of age and exposure to stories and screens is a better predictor of vocabulary size and explains a large portion of its variance. These findings stress out the strong effect of exposure variables on vocabulary size.

## 5. Conclusion

The purpose of the present study was to investigate the developmental trajectories of the vocabularies of bilingual children from diverse bilingual populations. This study has shown that English-Hebrew, French-Hebrew, and Russian-Hebrew speakers demonstrate similar vocabulary levels as well as balanced bilingualism at the group level. This study further validates the use of the multicultural questionnaire (Ohana and Armon-Lotem, 2023) with various bilingual populations and sets the ground for future research with larger samples. Future research might want to address some of the limitations of this study. First, the sample size is relatively small and so future studies should aim at collecting data from a larger sample. Second, in terms of language exposure of children to their two languages, two important notes should be considered. Information was obtained from parents in relation to the quantity of exposure as an estimated time period with no measure of the frequency of exposure. In addition, no information was received about the quality of exposure to each language. These variables might account for the individual variability observed in each group, and should be addressed in future studies. Despite these limitations, using the multicultural questionnaire is likely to enable researchers as well as health professionals to better assess the vocabularies of bilingual children from different linguistic background, as well as children who are exposed to each of their languages to a different extent.

## Data availability statement

The raw data supporting the conclusions of this article will be made available by the authors, without undue reservation.

## Ethics statement

The studies involving human participants were reviewed and approved by Bar Ilan University. Written informed consent to participate in this study was provided by the participants’ legal guardian/next of kin.

## Author contributions

OO and SA-L contributed to the design of the study and performed the statistical analysis. OO collected data and organized it and wrote the first draft of the manuscript. All authors contributed to the article and approved the submitted version.

## Funding

This research was supported by Bar-Ilan President’s Doctoral Fellowship of Excellence, Bar-Ilan Impact Center for Multilingualism and Multiculturalism across the Life Span (MultiLinC), and the Israel Science Foundation (Grant No. 454/18).

## Conflict of interest

The authors declare that the research was conducted in the absence of any commercial or financial relationships that could be construed as a potential conflict of interest.

## Editor’s note

Maria-José Ezeizabarrena edited the article in collaboration with Melita Kovacevic, University of Zagreb, Zagreb, Croatia.

## Publisher’s note

All claims expressed in this article are solely those of the authors and do not necessarily represent those of their affiliated organizations, or those of the publisher, the editors and the reviewers. Any product that may be evaluated in this article, or claim that may be made by its manufacturer, is not guaranteed or endorsed by the publisher.

## Footnotes

^1^www.bilingual-kids-israel.com
